# Pharmacogenetics and Gender Association with Psychotic Episodes on Nortriptyline Lower Doses: Patient Cases

**DOI:** 10.5402/2011/805983

**Published:** 2011-07-20

**Authors:** Irina Piatkov, Trudi Jones

**Affiliations:** ^1^Diversity Health Institute, Western Sydney Local Health District, North Parramatta, NSW 2151, Australia; ^2^DHI Lab, ICPMR Building, Westmead Hospital, Level 2 Westmead, NSW 2145, Australia

## Abstract

The variation in individual responses to psychotropic drug treatment remains a critical problem in the management of psychotic disorders. Although most patients will experience remission, some patients may develop drug-induced adverse effects that may range from troublesome to life threatening. Antidepressants are freely prescribed by general practitioners, and there should be constant awareness in the medical community about possible serious side effects. We describe two cases of adverse drug reactions on low dosage treatment that led to extreme psychotic episodes as examples of the potential for dangerous side effects. The patients developed adverse reactions on the normal recommended dosage of nortriptyline, a tricyclics antidepressant (TCA). Both were females, with no history of antidepressant treatment, unsocial behaviour, nor any family history of psychosis, but both experienced severe psychiatric symptoms. Pharmacogenetic tests can easily be performed and interpreted according to the likelihood of adverse reactions and should be included in toxicity interpretation.

## 1. Introduction

Two patients experienced single events of violent psychosis after the tricyclic antidepressant nortriptyline was prescribed in low dosages. Both patients were female, one Caucasian and the other Asian. Both were drug naïve with no history of any prescription or illicit drug use prior to the initiation of treatment. Both patients had neither history of antisocial behaviour nor any family history of psychosis.

**Patient A**

Female, 40 years old, Caucasian, with no history of violent behaviour or psychotic disorders in her family. She suffered from stress-induced mood changes. Her doctor recognised depression and prescribed nortriptyline, 10 mg 4 times/day. According to her husband, the next day she started to behave abnormally, complaining that she was hearing strange voices. On the third day, she experienced a sever psychotic episode. As the patient herself described, she felt dizzy, disorientated and was suffering from delusions. Since withdrawal of the drug, she has not experienced another episode. Pharmacogenetic tests revealed a loss-of-function CYP2D6*4/*41 polymorphism.

**Patient B**

Female, 17 years old, Asian, with no history of violent behaviour or psychotic disorders in her family. She suffered from stress during her high school exams. Depression was recognised by her doctor and nortriptyline was prescribed, 10 mg 3 times/day. After two weeks, she was admitted to the hospital with a severe psychotic episode. Since withdrawal of the drug, she has not experienced another episode. Pharmacogenetic tests revealed a CYP2D6*10/*10 polymorphism with diminished enzyme activity.

Epidemiological studies suggest that depression is the second most significant cause of disability in the health care arena. Up to 10% of the population at any one point in time may be depressed and up to 45% of the population may, at some point during their lifetime, suffer from a depressive episode [1]. The prescription of antidepressants has increased rapidly in recent years.

Tricyclic antidepressants (TCAs) are an older class of medication used for mood disorders and major depression. The mechanism of action is through blocking of the neuronal uptake of norepinephrine, serotonin, and dopamine. Anti-cholinergic, adrenergic and alpha-blocking actions of TCAs contribute to various side effects. TCAs have a very lipophilic nature and exhibit significant binding to proteins. This can be problematic in a case of overdose because both forced diuresis and hemodialysis are not helpful in eliminating the drug from the system [2]. 

TCAs are high-clearance drugs that are metabolised via multiple pathways involving both phase I (P450) and phase II (glucuronidation) processes. Elimination is dependent on hepatic hydroxylation via the cytochrome P450 mixed-function oxidase system, especially CYP2D6 [3–6], and conjugation with glucuronic acid [7]. 

Drugs which are the substrates for CYP2D6 are known to interfere with TCA metabolism [8–11], confirming the importance of CYP2D6 in nortriptyline metabolism. A significant correlation between amitriptyline clearance and the debrisoquine metabolic ratio has been observed in non-smokers [4], supporting some CYP2D6 dependence. 

**Figure 1:**
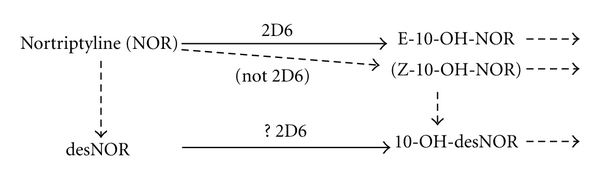
Nortriptyline metabolism.

Tricyclic antidepressants have a moderate therapeutic index, as they produce significant adverse effects at therapeutic concentrations and are dangerous in overdose. Significant side effects of TCAs are common and their prevalence is estimated as high as 5%, while acute poisoning with TCAs is potentially life threatening [12]. Central nervous system manifestations of toxicity include agitation, stupor, coma, seizure, and manic excitement. The plasma level correlates poorly with the severity of symptoms, and peak blood levels over 1000 ng/mL have a higher risk of cardiac and CNS toxicity [13, 14]. TCAs exist as tertiary or secondary amines and the tertiary forms are metabolised to secondary amines. Both tertiary and secondary amines are active, as are some of the subsequent hydroxylated metabolites. The tertiary amines are metabolised by many P450s, whereas the secondary amines are largely metabolised by CYP2D6 (Figure 1).

The major metabolite produced by CYP2D6, E-10-hydroxynortriptyline, has approximately half the potency of the parent drug in inhibiting noradrenaline reuptake and greatly reduced anticholinergic activity. It is often present at comparable (or higher) concentrations to the parent drug and may contribute to the antidepressant effects [15].

### 1.1. Genetic Polymorphisms

It is now recognised that genetic polymorphism differences in the metabolism of drugs can have a great influence on the efficacy and toxicity of medications. People with a particular genotype, or genetic characteristic, may suffer adverse responses to particular drugs, and such responses may be traced through families, ethnic groups, and geographic clusters. All pharmacogenetic polymorphisms, or relatively stable variations of the genes involved in drug metabolism, have been found to differ in frequency among some ethnic and racial groups. 

Cytochrome P450 2D6 is responsible for the metabolism of approximately 25% of prescription medicine. Many of these drugs are antipsychotic or antidepressant drugs, making it an important metabolic enzyme for psychiatry. Racial and ethnic studies of drug metabolism have shown substantial interpopulation differences in CYP2D6 enzyme activity. The activity of the CYP2D6 enzyme is extremely variable, due to the more than 50 recognised genetic variations, and it can be described as having four main levels of activity (http://www.cypalleles.ki.se/). The most important phenotype is the loss-of-function polymorphism or in some nomenclatures “poor metaboliser” (PM). The prevalence of loss-of-function polymorphisms in different ethnic groups has been extensively studied. In Caucasian populations, the prevalence of PMs is 5–10% [16–19]; in Asians—about 1% [20–24] and 0–19% in the African population [21–23, 25]. The prevalence of ultraextensive metabolisers is reported with a prevalence of 1.5–29% in different ethnic groups [26]. 

The ethnic variability in CYP2D6 activity can be attributed to genetic polymorphism of the CYP2D6 gene as a result of multiple mechanisms, including single point mutation, insertions or deletions, complete gene deletions, and gene duplication and multiplication. The major genetic polymorphisms associated with the poor metabolism phenotype lead to the complete lack of a functional protein.

The most common allele associated with poor metabolism is CYP2D6*4 which accounts for more than 70% of CYP2D6 alleles in loss-of-function polymorphism subjects [27]. Gough et al. [28] identified a G-to-A transition at the first nucleotide of exon 4 in the CYP2D6 gene, resulting in a shift of the splice site and introduction of a premature termination codon. The mutant protein had no residual activity. In DNA from an individual who was deficient in debrisoquine metabolism, Hanioka et al. [29] identified a 1934G-A transition (CYP2D6*4) at the junction of the third intron and fourth exon, resulting in an aberrant 3-prime splice recognition site and an mRNA with a single basepair deletion. The disrupted mRNA leads to a truncated protein without functional activity. They showed that the CYP2D6*4 homozygote or heterozygote state produces nonfunctional proteins, which is the cause of poor drug metabolism. CYP2D6 1934G>A (also seen as 1846G>A in the literature) is diagnostic for the nonfunctional CYP2D6*4 haplotype [30]. This variant is responsible for the majority of loss-of-function polymorphisms found in Caucasian populations [31] and is also found at much lower frequencies in other populations, such as Koreans [32]. 

CYP2D6 100C>T (also seen as 188C>T in the literature) is part of both the nonfunctional CYP2D6*4 haplotype and the reduced function CYP2D6*10 haplotype. According to Gaedigk et al. [33], the presence of CYP2D6 100C>T (188C>T) is diagnostic of CYP2D6*10. In vitro studies in both COS-1 [34] and V79 [35] cells have shown that cells transfected with CYP2D6 100C>T alone exhibit reduced function, suggesting that this mutation contributes to the reduced function of the CYP2D6*10 allele. Some attempts have been made to find an association of this polymorphism with generalised tonic clonic seizures, as seen in epilepsy [36] and tardive dyskinesia, in Chinese schizophrenic patients [37].

### 1.2. Dysfunctional CY2D6 Alleles and TCA Metabolism

In 1998 Dalén et al. [38] reported 3.3-, 2.8-, and 0.7-fold differences in the mean nortriptyline clearance between the groups of different CYP2D6 genotypes in Caucasians. Changes in clearance have also been seen with reduced activity alleles, including CYP2D6*10, in Asian subjects [39, 40]. 

Steimer et al. [41] demonstrated that dysfunctional CYP2D6 alleles had a greater risk of side effects with amitriptyline, 150 mg daily, than those with two functional alleles (77% versus 12%), and this risk was associated with higher nortriptyline concentrations.

Dizziness and sedation have been described with elevated nortriptyline concentrations in patients with a loss-of-function CYP2D6 genotype and individuals receiving CYP2D6 inhibitors [42, 43]. It was also shown that patients with ultrahigh activity of CYP2D6 showed treatment resistance [44]. 

Clinical manifestation of neurotoxicity is very complex and not clearly defined. Furthermore, all factors should be included in the evaluation of toxicity. It is not a surprise that clinical symptoms, like salivation, accommodation, sedation, blood pressure, and pulse rate did not vary between genotypes in a single-dose study in healthy subjects nor of patients with depression [38]. Also some results suggested that CYP2D6 polymorphisms did not influence the rate of adverse reactions to nortriptyline; however, with only 10 poor metabolisers (and the involvement of two drugs), the study was underpowered to detect moderate or small differences [45]. 

As the main nortriptyline metabolite is active, altered parent/metabolite ratios may be the reason that altered effects have been difficult to demonstrate; that is, there is minimal change in overall molar activity. It is notable that the major metabolite does not seem to have been measured in most of the assessments and is not considered clinically during therapeutic drug monitoring.

## 2. Method

Blood samples were referred to our laboratory by clinical doctors. Genotyping for CYP2D6 has been described in our previous publication in Pharmacogenomics [46]. 

## 3. Results

Patient A genotyping results revealed Cytochrome P450 polymorphism CYP2D6*4/*41.

Patient B genotyping results revealed Cytochrome P450 polymorphism CYP2D6*10/*10.

## 4. Discussion

Many factors can influence the efficacies and adverse reactions of medications, including age, gender, organ function (in particular the liver and kidneys), drug interactions, the nature and severity of the disease intended to treat, and the presence of other diseases. Nevertheless, genetic polymorphisms and gender account for the main variability in drug metabolism.

### 4.1. Cytochrome P450 Genetic Polymorphisms

Molecular genetics provides a practical approach to identifying biological predictors of psychotropic drug responses and drug-induced adverse events. A medication that is safe and effective in one patient may be ineffective or even harmful in another. The identification of relevant gene polymorphisms in patients prior to drug prescription will allow physicians to customise the selection of medication to meet individual patient needs.

As described earlier, several pharmacogenetic studies have focused on polymorphisms in liver cytochrome P450 isoenzymes which metabolise many antidepressant medications. The most intensively investigated gene is CYP2D6 and the CYP2D6 genotype has been shown to predict tricyclics concentrations in blood. 

The Caucasian patient described in this publication, who had a CYP2D6*4/*41 polymorphism with loss-of-function enzyme activity, exhibited her psychotic episode on the third day after treatment initiation. In contrast, the Asian patient with CYP2D6*10*10 polymorphism, with partially decreased enzyme activity, developed neurotoxicity syndrome two weeks after treatment initiation. This is consistent with current pharmacogenetic knowledge of CYP2D6 loss-of function polymorphisms.

### 4.2. Gender Differences in Response to Antidepressant

Gender differences in the diagnosis and treatment of mental disorders are described in numerous publications. 

It was shown that women appeared to have different plasma concentrations of antidepressants during treatment than men [47–50].

Yue et al. [40] pointed out, for example, that the monthly alterations in hormones in women may act on dopamine receptors in a protective manner, much like neuroleptics, and women require lower doses of psychotropics. McEwan [51] showed that antidepressants and neuroleptics have differing actions in men and women because of the effects of estrogen and testosterone in the brain. 

Furthermore, medications act differently in women before and after menopause and some researches suggest the effects of the menstrual cycle on pharmacokinetics [52–55].

It is also known that adipose tissue and body fluid quantities affect drug dosage and rate of metabolism and should be considered in the prescription of drug treatments. Pregnancy and menopause also affect the concentration of plasma proteins, and thus of protein-bound drugs [56]. 

Overall, women may experience more side effects or toxicity from the same dosage of a drug than do men.

## 5. Conclusion

Drug toxicology evidence is currently based on the drug to metabolite ratio in blood or urine. However, measurement of metabolites and their contribution to the validation of neurotoxicity remains uncertain due to the methodology of detection and lack of scientifically based evidence. In addition, each person is unique in his/her susceptibility to toxic agents. 

Interactions between substances metabolised through the Phase I cytochrome P450 system and an individual's variation in enzyme activity should be included in the assessment of toxicity. The described patient cases are illustrations of the value of individual interpretation of diminished Phase 1 metabolism and its consequences. 

There are often severe effects arising from the toxicity of antipsychotic medication. However, pharmacogenetic testing will help to avoid adverse reactions by providing information about an individual's drug metabolising capacity, thereby identifying the patients for whom a drug would be safe and effective. Phase 1 cytochrome P450 loss-of-function polymorphisms should be included in patient record warning labels, similar to allergies, as a warning that an individual patient is susceptible to particular adverse drug reactions.
